# The Psychological Impact of the COVID-19 Pandemic on Dentists in Germany

**DOI:** 10.3390/jcm10051008

**Published:** 2021-03-02

**Authors:** Mohamed Mekhemar, Sameh Attia, Christof Dörfer, Jonas Conrad

**Affiliations:** 1 Clinic for Conservative Dentistry and Periodontology, School of Dental Medicine, Kiel University, Arnold-Heller-Str. 3, Haus B, 24105 Kiel, Germany; doerfer@konspar.uni-kiel.de (C.D.); conrad@konspar.uni-kiel.de (J.C.); 2 Department of Cranio Maxillofacial Surgery, Justus-Liebig University Giessen, Klinik Str. 33, 35392 Giessen, Germany; sameh.attia@dentist.med.uni-giessen.de

**Keywords:** COVID-19, dentistry, IES-R, DASS-21, stress, anxiety, depression, dentists, psychological impact

## Abstract

Since the announcement of the coronavirus 2019 (COVID-19) outbreak as a pandemic, several studies reported increased psychological distress among healthcare workers. In this investigation, we examined the association between psychological outcomes and various factors among German dentists. Dentists from all German federal states were invited to participate in this study through a self-administered online questionnaire between July and November 2020. This questionnaire collected information on demographics, Depression Anxiety Stress Scales (DASS-21), and the Impact of Events Scale-Revised (IES-R) instrument. The associations displayed between demographic and psychological outcomes of depression, anxiety, stress, intrusion, avoidance, and hyperarousal were evaluated. Seven-hundred-and-thirty-two dentists participated in the survey and reported overall scores of (4.88 ± 4.85), (2.88 ± 3.57), (7.08 ± 5.04), (9.12 ± 8.44), (10.68 ± 8.88) and (10.35 ± 8.68) for depression, anxiety, stress, intrusion, avoidance, and hyperarousal, respectively. For females, being between 50–59 years of age, being immune deficient or chronically ill, working at a dental practice, and considering the COVID-19 pandemic a financial hazard were reported as significant associated factors (*p* < 0.05) with higher DASS-21 and IES-R scores. These findings underline the aspects which need to be taken into attention to protect the mental wellbeing of dentists in Germany during the crisis.

## 1. Introduction

Since the beginning of January 2020, COVID-19, a new contagious disease, has been threatening the health and welfare of humans globally. The viral pandemic was first defined in the Chinese city of Wuhan and was able to spread internationally in a few months. This rapid disease transmission with growing numbers of infected cases and associated critical health conditions or fatalities led to noticeable public anxiety and panic. Early studies examining immediate psychological impacts during the first COVID-19 wave of infection described moderate or severe psychological effects of the outbreak on the general population [1].

In addition to the psychological effects of the pandemic on the general population, healthcare workers are exposed to additional psychological difficulties due to their direct treatment of infected patients and the accompanying, increased risk of infection [2]. These include the fear of transmitting the disease to their families or loved ones [3], feeling discriminated against or rejected by society as potential carriers of the virus [4], as well as heavy workloads and time pressure, despite depleted personnel protection equipment [5].

Among all healthcare workers, the COVID-19 outbreak also negatively obstructed the activities of the dental profession [6,7,8]. Routine measures and dental treatments have been postponed due to the high risk of cross-infection during dental procedures [9,10,11,12]. Furthermore, oral mucosa has been described as a potential route of viral entry [13], restricting dental procedures to emergency treatments only to minimize the possible droplet infection. Dental manufacturers, companies, and some practices additionally suspended parts of their staff to counteract the financial complications during the pandemic [14]. Previous studies correspondingly described how dental professionals sense their moral responsibility to reduce their regular work to evade the cross-infection among their patients and relatives while having major concerns about the financial consequences of a lockdown or decreased patient visits [15]. Other investigations declared suspended research or educational activities [15], potential feelings of guilt among oral healthcare professionals, and scarce personal protective equipment [16] as possible causes of psychological distress among dentists during the worldwide outbreak [15].

To date, Germany has registered over one million SARS-CoV-2 infections, causing numerous fatalities related to COVID-19 health complications [17,18]. Federal states that are predominantly affected with high numbers of cumulative incidence (CI-cases per 100,000 residents) include Bavaria (CI = 1041), Baden-Württemberg, and (CI = 922) Saarland (CI = 875) [19]. In Germany, the distribution of the pandemic varies extensively across all locations of the country and is subject to dynamic change. Following the first outbreak caused by an infected traveler at the beginning of the year 2020 [20], further transborder contagions were mostly due to individuals returning from ski resorts in Italy and Austria, while local hotspots of infection have often been associated with crowded events such as carnivals or concerts [17]. Other settings related to high rates of infection within the German population were linked to working conditions, including crowded collective accommodations and workplaces [17], or working in close contact with infected individuals, as in the dental profession [21].

Germany is considered the largest member state of the European Union in terms of general population and number of oral healthcare professionals, with about 80.5 million inhabitants and nearly 70,000 dentists [22]. Under a scheme of statuary or private health insurance, general dental practitioners and dental specialists provide oral healthcare services to their patients in private practices or university clinics [23]. Although previous studies reported the financial burden affecting dental personnel in Germany during the COVID-19 pandemic [24], the psychological impact on German dentists, associated with the pandemic and its related factors, still needs to be unveiled. Therefore, this study aimed to investigate this topic using the Impact of Event Scale-Revised (IES-R) and Depression, Anxiety Stress Scale (DASS-21) surveys on a nationwide level among German dentists.

## 2. Materials and Methods

### 2.1. Study Population and Procedures

A nationwide cross-sectional survey was designed to evaluate the psychological impact of the COVID-19 pandemic and its related factors on German dentists. An online survey was created using a web-based survey tool (Unipark, QuestBack GmbH, Cologne, Germany) to diminish face to face communications and to allow easy participation for all dentists. After the approval by the University of Kiel Ethics Board (D452/18), the survey link was shared on various dental social network groups from different specialties, by different dental websites, magazines, and publishing companies, and was sent by email to registered dentists of different dental societies in Germany. The introductory text in the survey briefly clarified the research project and guaranteed anonymity and voluntary participation to the dentists. No financial incentives were promised to the contributors and no criteria of exclusion were defined (e.g., age, gender, or nationality). All participants consented at the beginning of the survey, confirming their readiness to contribute to the questionnaire. Data was collected between July 2020 and November 2020.

### 2.2. Survey Instruments

In the first part of the questionnaire, sociodemographic information was gathered on age, gender, federal state, marital status, number of children, workplace, comorbid medical diseases, and smoking status of the respondents. Participants were also asked whether they consider COVID-19 a personal financial threat to them or not.

In the second part of the survey, the Depression Anxiety Stress Scale (DASS-21) was provided to the participants. The Depression Anxiety Stress Scale (DASS-21) is a self-report instrument comprising 21 items that evaluate three psychological constructs: depression, anxiety, and stress [25,26]. Each subscale contains 7 statements that refer to the week before survey participation. Participants are asked to read the statements and rate them emotionally. Ratings are provided on a series of 4-point Likert-type scales from 0 (did not apply to me at all/ never) to 3 (applied to me very much/ always). Higher scores designate increased emotional and psychological distress. As previously described [27], the DASS-21 subscales were scored as follows: normal (0–4 DASS-21 points), mild (5–6 DASS-21 points), moderate (7–10 DASS-21 points), severe (11–13 DASS-21 points), and extremely severe (14+ DASS-21 points) for depression; normal (0–3 DASS-21 points), mild (4–5 DASS-21 points), moderate (6–7 DASS-21 points), severe (8–9 DASS-21 points), and extremely severe (10+ DASS-21 points) for anxiety; and normal (0–7 DASS-21 points), mild (8–9 DASS-21 points), moderate (10–12 DASS-21 points), severe (13–16 DASS-21 points), and extremely severe (17+ DASS-21 points) for stress. The German version of the DASS-21 survey was applied previously in several studies and showed good validity and reliability (78–91%) in the assessment of depression, anxiety, and stress levels [28,29].

The psychological impact of the outbreak was further assessed using the Impact of Event Scale-Revised (IES-R) tool [30,31], which is a validated 22-item self-assessment measuring the subjective psychological distress triggered by traumatic events. This assessment has 3 subscales (Intrusion, Avoidance, and Hyperarousal), which show close associations to symptoms of post-traumatic stress disorder. Respondents were requested to rate the distress level for each statement on similar Likert-type scales, also referring to the previous seven days of their survey. The IES-R subscores were categorized similar to previous investigations [31] as normal (0–23 IES-R points), mild (24–32 IES-R points), moderate (33–36 IES-R points), and severe psychological impact of events (>37 IES-R points) [30,32,33]. The German version of the IES-R survey was applied previously in several studies and presented good validity and reliability (79–90%) in the evaluation of the psychological impact of events [33,34,35,36]. Both IES-R and DASS-21 scales have been validated for use in recent investigations exploring the psychological impact of the COVID-19 outbreak on the general population and healthcare workers [1,31].

### 2.3. Sample Size Calculation

To determine the number of responding dentists needed for nationwide significant sample size, the following circumstances were defined for the sample size calculation:

1. The number of dentists in Germany (*n =* 70,000).
2. A confidence level of 95%.
3. A margin of error of 5%.

Based on these conditions it was determined that from all German federal states at least 383 dentists were needed for a statistically significant sample size.

### 2.4. Statistical Analysis

Data from the online questionnaire was digitally recorded by the web-based survey tool and exported afterwards for statistical analysis using SPSS software (SPSS Statistic 27, IBM, Armonk, NY, USA). Descriptive data analysis was performed on each question separately and the Shapiro–Wilk-Test was performed to test for normality of the data. Data were not normally distributed. Univariate analyses (Kruskal-Wallis and Mann-Whitney U test) were conducted to explore the associations between DASS-21/IES-R ratings and sociodemographic characteristics. In case of a significant test result post hoc, single comparisons were performed using the Dunn-Bonferroni test. Subsequently, multiple linear regression analyses were performed on DASS-21 total and subscores, as well as the IES-R subscales to identify the input of these previously identified, relevant factors. Statistical significance was set at *p* < 0.05.

## 3. Results

### 3.1. Participation and Sociodemographic Data

A total of 732 dentists participated in the survey resulting in a statistically significant sample. Participants included female (59.7%), male (40%), and third gender (0.3%) dentists from all federal states of Germany except Bremen. Almost half of the participants (53.3%) were 18–49 years old, while the other respondents were either 50–59 (31.6%) or over 60 (15.2%) years old. The majority of the contributing dentists were married or in a marriage-like relationship (82.5%) and had children (66.9%), while other participants were single (12.3%), divorced, separated, or widowed (5.2%) and had no children (33.1%). Nearly all dentists were working in private practices (95.4%), while a minority stated to work at a university clinic (2.9%) or in other institutions (1.6%). Among the respondents, around two-thirds considered the COVID-19 outbreak to be a threat to their financial security (61.3%). Moreover, the study population showed a smoking rate of 8.5% and different conditions of medical comorbidity with the highest being cardiovascular diseases (13.9%) and immunodeficiencies (4.8%) (Table 1).

### 3.2. DASS-21 and IES-R Scales and Associated Factors

The findings of the analysis for psychiatric symptoms in the overall sample according to the DASS-21 and IES-R scales were presented in (Table 2 and Table 3 and in association with the related factors in (Table 4 and Table 5), respectively.

The total study population presented DASS-21 and IES-R scores of normal psychological behaviors with potential mild distress due to the COVID-19 pandemic (Table 2 and Table 3), according to the applied scoring system.

DASS-21 and IES-R scales associated factors showed significantly higher DASS-21 and IES-R total and subscale scores denoting normal or mild psychological impact on depression, anxiety, stress, intrusion, avoidance, and hyperarousal among participating females, dentists working at private practices, or having systemic diseases, as well as among the respondents considering COVID-19 to be a financial threat (Table 4 and Table 5). Furthermore, the youngest and oldest groups of the participants (18–49 and ≥60 years) showed significantly lower DASS-21 and IES-R scores in comparison to the middle-aged group (50–59 years) of the survey (Table 4 and Table 5).

Multiple regression analyses of DASS-21 total and sub-scores within the study model showed a significant impact of financial factors, systemic immunodeficiency diseases, and age of the participants on the psychological stress, depression, and anxiety of German dentists (Table 6). Similarly, multiple regression analyses of IES-R scores displayed significant effects of analogous factors besides gender on intrusion, avoidance, and hyperarousal of the study participants (Table 7).

## 4. Discussion

In the early months of 2020, the first reported case of COVID-19 in Bavaria, Germany was confirmed [20]. Similar to the reactions globally, fast conversion and adaptation procedures were initiated in the healthcare system and instant steps were taken to counteract the outbreak. This severe and extraordinary crisis undoubtedly had an inevitable impact on healthcare workers nationwide. Among all healthcare divisions, the dental sector is considered highly distressed by the viral outbreak in Germany and worldwide due to various factors affecting the psychological steadiness and financial stability of dentists during the pandemic and its related lockdowns [6,26,37]. To date, this study is the first to evaluate the psychological effects of the COVID-19 pandemic on dentists in Germany nationwide.

In this survey 732 dentists participated via the online survey link and completed the online questionnaire, displaying a significant sample size and representing German dentists in the investigation. Sociodemographic data of the participants presented an analogous gender distribution compared to the dental population in Germany (60–70% female) and Europe (Table 1) with higher percentages of female dentists [23,38]. The majority of the survey participants were younger than 50 years old and working in dental practices (Table 1). This corresponds to the reported average age (48 years old) and equivalent professional characteristics among dentists in Germany [23,24,39]. Moreover, survey respondents displayed a smoking rate below 10% (Table 1) comparable to reported results of oral health professionals in Germany (5–8%), as well as in other dental communities worldwide [38,40]. Cardiovascular diseases exhibited the most prevalent systemic diseases among participating dentists, with a rate of 13.9% (Table 1), corresponding to the rates reported by previous studies on the German population (10–13%) [41].

In the current investigation, German dentists displayed overall mild psychological impact of the COVID-19 outbreak in terms of stress, anxiety, depression, intrusion, avoidance, and hyperarousal as estimated by the DASS-21 and IES-R survey systems. This presents obvious differences to healthcare professionals having higher levels of psychological distress in other countries worldwide [10,26,31,42,43,44] and might reflect the psychological value of Germany’s reported success to contain the infection rates of COVID-19, stabilize the financial state of its population during the crisis and communicate the rationale for its policies to cope with the emergency [45,46,47]. Corresponding to the described high rates of infection and cumulative incidence of COVID-19 in different German federal states [17], several regions such as Saarland and other southern states displayed higher scores of psychological impacts on DASS-21 and IES-R scales (Table 4 and Table 5). Furthermore, multiple associated factors seem to play an effective role in the amount of stress, anxiety, and depression, besides potential symptoms of PTSD affecting dental professionals in Germany during the crisis. Similar to previous investigations on healthcare workers and dentists, female participants in the current study exhibited significantly higher scores of anxiety, stress, and all three IES-R subscales (Table 4 and Table 5) [26,48] than their male counterparts. This might be related to the fact that females generally show significantly greater risks than males to develop anxiety, stress, and depressive disorders, as well as PTSD symptoms during adulthood [49,50]. This difference was explained as being due to discrepant thought control strategies and metacognitive beliefs between genders, leading to more emotional and neurotic distress among females [50]. Although only 0.3% of the survey respondents represented the third gender (Table 1), these participants revealed very high scores of psychological distresses in comparison to both males and females (Table 4 and Table 5). This result also confirms the previously described depressive symptoms, interpersonal trauma exposure, stress, anxiety, and general distress among transgender and gender non-conforming populations due to minority stress processes and multiple social factors [51]. The current investigation further showed that being single, married, or in a marriage-like relationship and having children among the participants were associated with lower total DASS-21 and IES-R scores and sub-scores compared to being divorced, separated, or widowed (Table 4 and Table 5) as well as having no children. As defined by earlier studies, this observation was similar in other countries among healthcare workers [26,52] and might be due to the reported lower levels of stress and psychological disorders of couples in a relationship in comparison to divorced or widowed individuals. Moreover, intimate and family relationships facilitate dyadic coping and social support to help as a buffer against difficult situations, which can then translate into lower levels of psychological distress [53,54]. In the current survey, the age factor also demonstrated a significant role in the determination of dentists’ psychological status or levels of mental tension in association with the COVID-19 outbreak. As observed in the results, respondents of the youngest age group (18–49 years) and the group over 60 years had overall lower DASS-21 and IES-R scores than the middle-aged group (50–59 years) with significant DASS-21 total, DASS-21 stress, and IES-R intrusion, as well as hyperarousal outcomes (Table 4 and Table 5). As the Robert-Koch Institute, the main German federal government agency and research institute responsible for disease control and prevention, stated officially that the risk of severe COVID-19 complications and mortality increases steadily from 50 to 60 years of age [55], this aspect might increase the psychological burden upon the middle-aged group (50–59 years) due to their fear of death, illness or complications [56,57]. Moreover, older participants might have further health-associated age-dependent risk factors [55], which can increase the possibility of getting infected by COVID-19 or having dangerous health complications as medically compromised patients. Interestingly, the oldest age group (over 60 years old) of the study showed even lower DASS-21 and IES-R scores than the youngest participants (Table 4 and Table 5). This finding that people aged 60 years and above displayed less psychological distress on this investigation’s rating scales is very thought-provoking, as COVID-19 infections have been shown to cause significantly higher morbidity and mortality in this age group in comparison with younger individuals [58,59]. Since the media and health organizations emphasized the need for people over 60 years particularly to perform strict procedures of social distancing, as they are more likely to have underlying medical risk conditions, it may have been anticipated that older participants would be more psychologically affected during the pandemic. Nevertheless, this outcome is consistent with some previous investigations that described decreased indicator scores in stress, anxiety, and depression of younger individuals in comparison to older age groups in European and North American countries [60,61]. This observed psychological stability of the older group could be explained by the fact that many respondents of this group might be retired from the dental career, as the general age of retirement in Germany starts with 65 years [62]. Being in distance from patient treatment as a dentist during the COVID-19 crisis could eliminate multiple factors provoking the mental tension during the pandemic, as the stress and fear of getting infected during treatment or taking the infection to family members [10], the anxiety of treating patients with suspicious symptoms [6,16], or the depression and distress of losing the job and financial safety [24]. Moreover, older people incline to show less social mobility than younger individuals, which could explain their lower stress, anxiety, and depression during a pandemic lockdown [61]. People above 60 years are correspondingly expected to have experienced numerous difficult major life events in their past, such as war, past pandemics, or financial crises, therefore increasing their resilience, as observed in the current study [61]. Another theory that could be advocated to clarify this outcome is that older people usually spend less time on social media due to their frequent resistance to social networking sites [63]. High rates of news consumption about the pandemic have been linked previously with elevated levels of psychological distress [64].

The presence of medical comorbidities during the COVID-19 pandemic has often been linked to an increase in in-hospital complications and mortality rates [65,66]. Congruently, individuals with systemic diseases and medical risk-factors have reported higher rates of psychological pressure and distress [67]. According to this investigation, dentists in Germany have displayed the same significant inclination during the COVID-19 emergency among participants with chronic liver diseases or medical conditions of immune deficiency (Table 4). This outcome resembles previous findings of healthcare workers in other countries, where health professionals living among persons with immune deficiency or chronic diseases significantly reported higher stress and anxiety scores [68,69]. Indeed, both medical conditions are considered among the highest risk factors associated with severe medical complications after COVID-19 infection [70,71], which clarifies the reason for psychological distress.

Workplace-related stress, anxiety, and depression are among the most important factors affecting the mental health wellbeing of people worldwide [72]. In previous reports dentists and healthcare workers equally conveyed various stressors within their workplace including the risk of infection, constant time pressure, concern over their capability to provide dental or medical services in the future, and financial pressure [73,74]. These workplace stressors have significantly increased during the COVID-19 pandemic among dental professionals globally [26,74]. According to the current survey German dentists working in a private dental practice displayed significantly higher stress, hyperarousal, and intrusion scores on the DASS-21 and IES-R scales than their colleagues at university clinics (Table 4 and Table 5). This observed outcome could be explained by different aspects. While university dentists usually have their working-time divided into three branches comprising education, research, and patient treatment, dental practices follow a policy mostly aimed to treat the maximum number of patients. This may give university dentists a feeling of safety from exposure to infection through continuous patient treatment [74]. Furthermore, previous reports described the lack of protective equipment against the COVID-19 infection in German non-university health facilities, which can confidently increase the risk of personnel infection and its related psychological distress [75]. Having to face the lockdown and lack of patients during the pandemic as employers or employees of private dental practices, this situation created a financial crisis for many German dental practices and non-university health facilities [24,75], which is considered one of the main sources of psychological distresses among healthcare workers worldwide [76,77]. This was also confirmed by the current survey as participants considering the pandemic a financial threat reported significantly higher scores on both DASS-21 and IES-R scales in all parameters (Table 4 and Table 5). Notably, dentists who stated to work in other facilities (Table 4 and Table 5) also showed lower psychological distress than their colleagues in dental practices. This may be due to similar factors as those stated above, as they could working at non-clinical institutions as dental companies or manufacturers.

To ascertain the independent effects of the measured significant factors of COVID-19 being a financial threat, workplace, gender, age, and medical comorbidities on the DASS-21 and IES-R scores and subscores, multiple linear regression analyses were conducted. Female gender, an age between 50–59 years, being immune deficient, and considering the COVID-19 pandemic a financial hazard were independently associated with worse psychiatric outcomes (Table 4 and Table 5) marking these aspects as the most effective on German dentists and their mental health during the COVID-19 crisis.

## 5. Limitations

To the best of our knowledge, this investigation is the first one in Germany examining the psychological impact of COVID-19 on dentists nationwide. Nevertheless, we recognize some limitations to our study. First of all, the investigation is restricted by its cross-sectional nature and lacks the follow-up on a longitudinal level. On the other hand, misinterpretations in such investigations are known to be equally dispersed [23]. In this survey, the observed outcomes of German dentists cannot be accredited exclusively to the analyzed aspects and socio-environmental information. Additional co-variables and sociodemographic observations (including being an employer or employee, exact financial challenges, and the number of treated patients) could play an important role in changing some outcomes or interpretations of the study. The data collection phase of the study was completed within three months. Given the time-sensitivity throughout this crisis and rapid changes in regulations and infection rates, these aspects might also influence the results reported by the respondents. Furthermore, the voluntary nature of the investigation might have caused a selection bias among the German dentists. Finally, to reach the maximum number of participants and to diminish face to face conditions, we applied an online self-report questionnaire to assess psychological symptoms that do not rely on diagnostic evaluation by mental health professionals. Adding a clinical mental health evaluation by psychiatric specialists would definitely contribute to the outcome of the survey. Regardless of the above limitations, conclusions of this survey provide important information on the psychological impact of COVID-19 on dentists across Germany.

## 6. Conclusions

The mental wellness of dentists is vital for guaranteeing the sustainability of dental services during the struggle with the COVID-19 pandemic. Our findings among the study population display being female, in an age group between 50–59 years, being immune deficient or chronically ill, working at a dental practice, and considering the COVID-19 pandemic a financial hazard are significant factors which cause distress in German dentists during the COVID-19 crisis and reporting higher DASS-21 and IES-R scores and subscores. Analyzing these aspects can assist health authorities in Germany in implementing the needed actions to diminish the unwanted psychological effects of the pandemic and their influencing factors on the German dental community.

## Figures and Tables

**Table 1:** Characteristics of Participants (*n* = 732).

		*n*	%
**Gender**			
	Female	437	59.7
	Male	293	40
	Third Gender	2	0.3
**Age**			
	18–49	390	53.3
	50–59	231	31.6
	≥60	111	15.2
**Marital status**			
	Single	90	12.3
	Married or in a marriage-like partnership	604	82.5
	Divorced, separated, or widowed	38	5.2
**Having children**			
	Yes	490	66.9
	No	242	33.1
**COVID-19 being a personal financial threat**			
	Yes	449	61.3
	No	283	38.7
**Workplace** ^1^			
	Dental practice	698	95.4
	University clinic	21	2.9
	other	12	1.6
**Federal state**			
	Hamburg	12	1.6
	Mecklenburg-Western Pomerania	17	2.3
	Schleswig-Holstein	42	5.7
	Brandenburg	11	1.5
	Berlin	42	5.7
	Lower Saxony	32	4.4
	Baden-Württemberg	275	37.6
	Thuringia	7	1
	Hesse	37	5.1
	Saarland	7	1
	Bavaria	82	11.2
	Saxony-Anhalt	6	0.8
	Saxony	19	2.6
	North Rhine-Westphalia	117	16.0
	Rhineland-Palatinate	26	3.6
**Smoker**			
	Yes	62	8.5
	No	670	91.5
**Medical comorbidity** ^2^			
	Diseases of the cardiovascular system (e.g., coronary heart disease and high blood pressure)	102	13.9
	Chronic lung diseases (e.g., COPD)	19	2.6
	Chronic liver diseases	6	0.8
	Diabetes mellitus	12	1.6
	Cancer	18	2.5
	Immunodeficiency	35	4.8

^1^ Multiple choice was possible; ^2^ No or multiple choice was possible.

**Table 2:** DASS-21 and IES-R Scores of the Study Sample.

		Mean ± SD	Interquartile Range
**DASS-21** (*n* = 729) ^1^			
	Total	14.84 ± 12.31	17
	Depression	4.88 ± 4.85	6
	Anxiety	2.88 ± 3.57	5
	Stress	7.08 ± 5.04	7
**IES-R** (*n* = 727) ^1^			
	Intrusion	9.12 ± 8.44	13
	Avoidance	10.68 ± 8.88	14
	Hyperarousal	10.35 ± 8.68	13

^1^*n* varies because of missing data.

**Table 3:** Amount of dentists and total population percentage for each DASS-21 and IES-R subscale category.

	Subscale	Category	*n*	%
**DASS-21** (*n* = 729) ^1^				
	Depression	normal	413	56.7
		mild	105	14.4
		moderate	106	14.5
		severe	47	6.4
		extremely severe	58	8
	Anxiety	normal	506	69.4
		mild	90	12.3
		moderate	47	6.4
		severe	33	4.5
		extremely severe	53	7.3
	Stress	normal	427	58.6
		mild	86	11.8
		moderate	97	13.3
		severe	81	11.1
		extremely severe	38	5.2
**IES-R** (*n* = 727) ^1^				
	Intrusion	normal	679	93.3
		mild	43	5.9
		moderate	6	0.8
		severe	0	0
	Avoidance	normal	651	89.5
		mild	68	9.4
		moderate	7	1
		severe	1	0.1
	Hyperarousal	normal	665	91.3
		mild	54	7.4
		moderate	9	1.2
		severe	0	0

^1^*n* varies because of missing data.

**Table 4:** Differences between participants characteristics regarding DASS-21 total and subscale scores.

	DASS-21 Total			DASS-21 Depression			DASS-21 Anxiety			DASS-21 Stress		
	Mean ± SD	Test Statistic	*p*-Value	Mean ± SD	Test Statistic	*p*-Value	Mean ± SD	Test Statistic	*p*-Value	Mean ± SD	Test Statistic	*p*-Value
**Gender**												
Female	15.05 ± 11.86	H = 6.75	0.03	4.71 ± 4.63	H = 5.01	0.08	2.99 ± 3.50	H = 9.53	0.01	7.35± 4.95	H = 8.35	0.02
Male	14.35 ± 12.84			5.07 ± 5.10			2.67 ± 3.65			6.61 ± 5.11		
Third Gender	38.00 ± 7.07			15.00 ± 0			8.50 ± 0.70			14.50 ± 6.36		
Posthoc: Dunn-Bonferroni-Test												
Male-Female		23.85	0.13					36.11	0.02		36.40	0.02
Male-Third G		−330.39	0.03					−317.67	0.03		−284.25	0.06
Female-Third G		−306.54	0.04					−281.56	0.06		−247.85	0.10
**Age**												
18–49	14.70 ± 11.93	H = 7.45	0.02	4.71 ± 4.73	H = 4.23	0.12	2.86 ± 3.44	H = 4.01	0.14	7.13 ± 4.97	H = 10.59	0.01
50–59	16.16 ± 12.99			5.40 ± 5.12			3.17 ± 3.83			7.60 ± 5.16		
≥60	12.49 ± 11.91			4.36 ± 4.63			2.35 ± 3.41			5.78 ± 4.82		
Posthoc: Dunn-Bonferroni-Test												
≥60 - 18–49		48.04	0.11								61.40	0.02
≥60 - 50–59		66.62	0.02								78.44	0
18–49 - 50–59		−18.57	0.29								−17.05	0.99
**Marital status**												
Single	13.98 ± 11.65	H = 0.30	0.86	4.68 ± 4.60	H = 0.10	0.95	2.52 ± 3.29	H = 1.21	0.54	6.78 ± 4.86	0.29	0.87
Married or in a marriage-like partnership	14.91 ± 12.33			4.87 ± 4.84			2.93 ± 3.63			7.11 ± 5.03		
Divorced, separated, or widowed	15.70 ± 13.69			5.57 ± 5.59			2.89 ± 3.19			7.24 ± 5.66		
**Having children**												
Yes	14.52 ± 12.01	U = 60,853	0.47	4.63 ± 4.61	U = 63,074	0.12	2.84 ± 3.60	U = 60,514.50	0.54	7.04 ± 4.99	U = 59,231	0.91
No	15.47 ± 12.89			5.37 ± 5.27			2.95 ± 3.51			7.14 ± 5.14		
**COVID-19 being a personal financial threat**												
Yes	18.46 ± 12.68	U = 33,639	0	6.20 ± 5.08	U = 35,374.50	0	3.69 ± 3.87	U = 40,350	0	8.56 ± 5.05	U = 34,606.50	0
No	9.13 ± 9.15			2.79 ± 3.58			1.60 ± 2.56			4.74 ± 4.04		
**Workplace (Multiple Choice)**												
Dental practice	15.03 ± 12.24	U = 15,076	0.01	4.92 ± 4.81	U = 13,960	0.07	2.89 ± 3.54	U = 13,646	0.12	7.21 ± 5.04	U = 16,018	0
University clinic	6.29 ± 7.86	U = 3867	0	2.05 ± 2.77	U = 4652	0	1.57 ± 3.06	U = 5057	0.01	2.67 ± 2.42	U = 3289.50	0
other	16.75 ± 15.70	U = 4400.50	0.89	6.42 ± 6.64	U = 4842.50	0.45	3.75 ± 4.71	U = 4590	0.68	6.58 ± 5.35	U = 4069.50	0.74
**Federal state**												
Hamburg	12.17 ± 8.08	H = 18.38	0.19	4.50 ± 3.45	H = 13.79	0.47	2.08 ± 1.93	H = 20.93	0.10	5.58 ± 4.19	H = 23.02	0.06
Mecklenburg-Western Pomerania	9.82 ± 12.81			4.18 ± 5.33			1.53 ± 3.06			4.12 ± 4.91		
Schleswig-Holstein	11.90 ± 12.32			3.83 ± 4.49			2.51 ± 3.65			5.56 ± 5.09		
Brandenburg	16.09 ± 9.19			6.09 ± 4.13			2.45 ± 2.51			7.55 ± 3.42		
Berlin	13.40 ± 12.35			4.14 ± 4.31			2.74 ± 3.92			6.52 ± 4.94		
Lower Saxony	13.12 ± 12.71			4.97 ± 5.50			1.97 ± 3.47			6.19 ± 4.94		
Baden-Württemberg	15.30 ± 12.21			4.75 ± 4.68			2.96 ± 3.56			7.58 ± 5.09		
Thuringia	14.14 ± 17.83			4.29 ± 6.16			3.71 ± 4.79			6.14 ± 7.24		
Hesse	16.64 ± 12.82			5.89 ± 5.18			3.42 ± 3.64			7.33 ± 5.05		
Saarland	20.29 ± 22.77			7.43 ± 8.58			5.43 ± 7.79			7.43 ± 6.88		
Bavaria	14.21 ± 11.91			4.84 ± 5.01			2.79 ± 3.22			6.57 ± 4.97		
Saxony-Anhalt	18.00 ± 12.28			5.50 ± 4.59			3.67 ± 2.25			8.83 ± 6.15		
Saxony	12.00 ± 9.33			3.53 ± 3.79			2.11 ± 3.04			6.37 ± 3.83		
North Rhine-Westphalia	16.69 ± 12.47			5.55 ± 5.08			3.39 ± 3.64			7.75 ± 5.07		
Rhineland-Palatinate	14.19 ± 11.52			5.08 ± 4.93			2.08 ± 3.32			7.04 ± 4.37		
**Smoker**												
Yes	14.40 ± 12.17	U = 21,121	0.78	4.77 ± 4.56	U = 20,902.50	0.89	2.82 ± 3.80	U = 21,417	0.63	6.81 ± 4.86	U = 21,280	0.70
No	14.88 ± 12.33			4.89 ± 4.88			2.89 ± 3.55			7.10 ± 5.05		
**Medical comorbidity (Multiple Choice)**												
No medical comorbidities	14.24 ± 12.07			4.65 ± 4.75			2.68 ± 3.44			6.90 ± 5.03		
Diseases of the cardiovascular system (e.g., coronary heart disease and high blood pressure)	16.75 ± 13.43	U = 34,782	0.16	5.64 ± 5.27	U = 34,684.50	0.17	3.63 ± 4.08	U = 35,670.50	0.06	7.49 ± 5.18	U = 33,574.50	0.42
Chronic lung diseases (e.g., COPD)	14.11 ± 9.53	U = 6882.50	0.88	4.26 ± 3.54	U = 6628	0.90	2.95 ± 2.92	U = 7072.50	0.71	6.89 ± 4.38	U = 6811.50	0.94
Chronic liver diseases	22.50 ± 12.19	U = 2999.50	0.11	8.83 ± 4.22	U = 3303	0.03	4.33 ± 4.13	U = 2611	0.39	9.33 ± 4.76	U = 2786	0.23
Diabetes mellitus	19.17 ± 14.03	U = 5115.50	0.26	7.00 ± 5.86	U = 5180.50	0.22	3.17 ± 3.33	U = 4650.50	0.62	9.00 ± 6.27	U = 4978.50	0.35
Cancer	13.67 ± 10.45	U = 6291	0.90	4.44 ± 3.62	U = 6478.50	0.93	2.56 ± 3.52	U = 6206	0.82	6.67 ± 4.42	U = 6175.50	0.80
Immunodeficiency	19.14 ± 12.61	U = 15014	0.02	6.80 ± 5.21	U = 15,352.50	0.01	4.14 ± 4.02	U = 14791	0.03	8.20 ± 4.32	U = 14,088.50	0.11

SD = Standard deviation; H = Test statistic of Kruskal-Wallis test; U = Test statistic of Mann-Whitney U test; Significant result are highlighted.

**Table 5:** Differences between participants characteristics regarding IES-R subscale scores.

	IES-R Intrusion			IES-R Avoidance			IES-R Hyperarousal		
	Mean ± SD	Test Statistic	*p*-Value	Mean ± SD	Test Statistic	*p*-Value	Mean ± SD	Test Statistic	*p*-Value
**Gender**									
Female	9.74 ± 8.36	H = 13.18	0.001	11.74 ± 9.12	H = 17.51	0	10.87 ± 8.64	9.59	0.01
Male	8.12 ± 8.44			9.07 ± 8.26			9.48 ± 8.65		
Third Gender	20.00 ± 9.90			15.50 ± 12.02			22.50 ± 3.54		
Posthoc: Dunn-Bonferroni-Test									
Male-Female		51.60	0.001		65.27	0		39.10	0.01
Male-Third G		−271.90	0.07		−165.01	0.27		−304.00	0.04
Female-Third G		−220.30	0.14		−99.74	0.50		−264.90	0.08
**Age**									
18–49	8.59 ± 8.21	H = 6.78	0.03	10.69 ± 8.78	H = 4.52	0.10	9.88 ± 8.48	H = 9.76	0.01
50–59	10.38 ± 8.92			11.38 ± 9.14			11.78 ± 9.05		
≥60	8.35 ± 7.97			9.23 ± 8.60			9.04 ± 8.24		
Posthoc: Dunn-Bonferroni-Test									
≥60 - 18–49		0.62	0.98					19.53	0.39
≥60 - 50–59		44.07	0.07					65.57	0.02
18–49 - 50–59		−43.45	0.04					−46.04	0.03
**Marital status**									
Single	7.08 ± 7.32	H = 5.45	0.07	10.13 ± 8.63	H = 1.41	0.50	8.89 ± 7.50	H = 2.34	0.31
Married or in a marriage-like partnership	9.37 ± 8.46			10.85 ± 8.95			10.61 ± 8.82		
Divorced, separated, or widowed	10.03 ± 10.15			9.34 ± 8.38			9.76 ± 8.93		
**Having children**									
Yes	9.01 ± 8.38	U = 59,615	0.69	10.33 ± 8.62	U = 61,854	0.20	10.33 ± 8.58	U = 58,065	0.85
No	9.34 ± 8.57			11.41 ± 9.37			10.38 ± 8.89		
**COVID-19 being a personal financial threat**									
Yes	11.53 ± 8.69	U = 34,886.50	0	12.82 ± 9.00	U = 39,615	0	13.00 ± 8.83	U = 33,329	0
No	5.33 ± 6.41			7.34 ± 7.58			6.18 ± 6.55		
**Workplace (Multiple Choice)**									
Dental practice	9.26 ± 8.42	U = 14,933.50	0.01	10.79 ± 8.88	U = 13,820	0.09	10.54 ± 8.66	U = 15,584	0
University clinic	4.38 ± 6.31	U = 4531.50	0	7.43 ± 8.07	U = 5633	0.06	4.10 ± 5.22	U = 3917.50	0
other	6.50 ± 8.95	U = 3239	0.14	7.83 ± 8.64	U = 3388	0.21	8.58 ± 9.90	U = 3553.50	0.30
**Federal state**									
Hamburg	8.00 ± 7.84	H = 14.29	0.43	10.42 ± 7.23	H = 16.88	0.26	9.58 ± 7.85	H = 17.92	0.21
Mecklenburg-Western Pomerania	5.53 ± 6.75			6.24 ± 7.09			5.88 ± 7.80		
Schleswig-Holstein	8.43 ± 8.07			9.14 ± 7.05			8.71 ± 9.35		
Brandenburg	9.36 ± 10.31			10.18 ± 11.07			9.64 ± 8.63		
Berlin	8.83 ± 9.01			11.32 ± 9.94			9.24 ± 8.78		
Lower Saxony	10.75 ± 10.28			9.53 ± 7.83			8.66 ± 8.25		
Baden-Württemberg	9.31 ± 8.35			10.90 ± 9.15			11.00 ± 8.62		
Thuringia	6.29 ± 9.46			8. 71 ± 12.33			10.00 ± 11.21		
Hesse	9.76 ± 8.43			10.30 ± 7.54			10.03 ± 7.94		
Saarland	9.43 ± 9.61			7.57 ± 7.48			11.29 ± 12.45		
Bavaria	8.26 ± 8.28			10.35 ± 9.37			9.93 ± 8.77		
Saxony-Anhalt	7.83 ± 6.56			14.00 ± 5.73			13.33 ± 6.71		
Saxony	5.89 ± 7.21			7.84 ± 8.07			9.26 ± 8.68		
North Rhine-Westphalia	10.01 ± 8.62			12.14 ± 8.94			11.72 ± 9.07		
Rhineland-Palatinate	10.58 ± 7.51			12.35± 8.98			9.12 ± 6.44		
**Smoker**									
Yes	9.00 ± 8.34	U = 20,444	0.95	10.41 ± 8.38	U = 20,551.50	0.88	10.49 ± 8.28	U = 19,770.50	0.72
No	9.13 ± 8.46			10.71 ± 8.93			10.34 ± 8.72		
**Medical comorbidity (Multiple Choice)**									
No medical comorbidities	8.85 ± 8.29			10.77 ± 8.88			10.00 ± 8.55		
Diseases of the cardiovascular system (e.g., coronary heart disease and high blood pressure)	9.34 ± 8.76	U = 31,913	0.90	9.37 ± 8.14	U = 28,785	0.15	11.18 ± 9.10	U = 33,471	0.36
Chronic lung diseases (e.g., COPD)	10.84 ± 8.24	U = 7758.50	0.26	11.37 ± 9.35	U = 7073.50	0.70	9.95 ± 6.39	U = 6952.50	0.81
Chronic liver diseases	15.00 ± 10.47	U = 2909	0.15	15.67 ± 9.42	U = 2895.50	0.15	16.83 ± 8.52	U = 3140.50	0.06
Diabetes mellitus	9.50 ± 9.60	U = 4349.50	0.94	10.25 ± 9.10	U = 4180.50	0.88	12.67 ± 10.27	U = 4853.50	0.44
Cancer	11.44 ± 9.35	U = 7469	0.22	10.83 ± 7.58	U = 6770.50	0.66	12.11 ± 9.27	U = 7188.50	0.36
Immunodeficiency	11.34 ± 9.58	U = 13,991	0.12	13.23 ± 10.49	U = 13,822.50	0.16	13.20 ± 9.84	U = 14,320	0.07

SD = Standard deviation; H = Test statistic of Kruskal-Wallis test; U = Test statistic of Mann-Whitney U test; Significant result are highlighted.

**Table 6:** Multiple regression analyses with relevant factors of DASS-21 total and subscores.

	B	SE	β	T	*p*	95% CI
**DASS-21 Total**						
Gender ^1^	−0.04	0.88	−0	−0.04	0.97	−1.76; 1.69
Age ^2^	−0.87	0.60	−0.05	−1.45	0.15	−2.04; 0.31
COVID-19 being personal financial threat ^3^	−9.05	0.89	0.36	−10.20	0	−10.79; −7.31
Workplace: Dental practice ^4^	−1.74	3.21	−0.03	−0.54	0.59	−8.03; 4.56
Workplace: University clinic ^4^	−5.54	4.08	0.08	−1.36	0.18	−13.56; 2.48
Medical comorbidity: Immunodeficiency ^4^	3.94	1.98	0.07	1.99	0.05	0.05; 7.83
**DASS-21 Depression**						
COVID-19 being personal financial threat ^3^	−3.29	0.35	−0.33	−9.30	0	−3.98; −2.59
Workplace: University clinic ^4^	−0.98	1.03	0.03	−0.96	0.34	−3.00; 1.03
Medical comorbidity: Chronic liver diseases ^4^	2.76	1.87	0.05	1.48	0.14	−0.91; 6.43
Medical comorbidity: Immunodeficiency ^4^	1.79	0.79	0.08	2.27	0.02	0.24; 3.33
**DASS-21 Anxiety**						
Gender ^1^	−0.23	0.26	0.03	−0.92	0.36	−0.73; 0.27
COVID-19 being personal financial threat ^3^	−2.07	0.27	−0.28	−7.81	0	−2.59; −1.55
Workplace: University clinic^4^	−0.12	0.77	−0.01	−0.15	0.88	−1.63; 1.40
Medical comorbidity: Immunodeficiency ^4^	1.16	0.59	0.07	1.95	0.05	−0.01; 2.32
**DASS-21 Stress**						
Gender ^1^	−0.44	0.35	−0.04	−1.23	0.22	−1.14; 0.26
Age ^2^	−0.51	0.24	−0.07	−2.09	0.04	−0.99; −0.03
COVID-19 being personal financial threat ^3^	−3.70	0.36	−0.36	−10.25	0	−4.41; −2.99
Workplace: Dental practice ^4^	0.99	1.31	0.04	0.76	0.45	−1.57; 3.56
Workplace: University clinic ^4^	−1.54	1.66	−0.05	−0.92	0.36	−4.80; 1.73

B = unstandardized beta coefficient; SE = standard error; β = standardized beta coefficient; *p* = *p*-value; CI: confidence interval; Significant results are highlighted; ^1^ 1 = female; 2 = male; 3 = third gender; ^2^ 1 = 18–49 years; 2 = 50–59 years; 3 = ≥ 60 years; ^3^ 1 = yes; 2 = no; ^4^ 0 = not quoted; 1 = quoted.

**Table 7:** Multiple regression analyses with relevant factors of IES-R scores.

IES-R Intrusion						
Gender ^1^	−1.57	0.61	−0.09	2.59	0.01	−2.76; −0.38
Age ^2^	0.35	0.41	0.03	0.86	0.39	−0.45; 1.16
COVID-19 being personal financial threat ^3^	−6.14	0.61	−0.36	−10.05	0	−7.34; −4.94
Workplace: Dental practice ^4^	0.67	2.21	0.02	0.30	0.76	−3.67; 5
Workplace: University clinic ^4^	−0.35	2.81	−0.01	−0.12	0.90	−5.87; 5.17
**IES-R Avoidance**						
Gender ^1^	−2.59	0.63	−0.15	−4.13	0	−3.82; −1.36
COVID-19 being personal financial threat ^3^	−5.52	0.34	0.30	8.65	0	−6.77; −4.27
**IES-R Hyperarousal**						
Gender ^1^	−1.21	0.62	−0.07	−1.96	0.05	−2.42; 0
Age ^2^	0.04	0.42	0	0.10	0.92	−0.78; 0.86
COVID-19 being personal financial threat ^3^	−6.68	0.62	−0.38	−10.74	0	−0.79; −5.46
Workplace: Dental practice ^4^	1.20	2.25	0.03	0.53	0.59	−3.22; 5.61
Workplace: University clinic ^4^	−1.22	2.86	−0.02	0.43	0.67	−6.84; 4.40

B = unstandardized beta coefficient; SE = standard error; β = standardized beta coefficient; p = *p*-value; CI: confidence interval; Significant results are highlighted; ^1^ 1 = female; 2 = male; 3 = third gender; ^2^ 1 = 18−49 years; 2 = 50−59 years; 3 = ≥ 60 years; ^3^ 1 = yes; 2 = no; ^4^ 0 = not quoted; 1 = quoted.

## Data Availability

The data presented in this study are available on request from the corresponding author.
